# The efficacy of combination therapy with Ningmitai capsule and sildenafil in men with chronic prostatitis/chronic pelvic pain syndrome and erectile dysfunction: a prospective, multicenter, randomized controlled trial

**DOI:** 10.1093/sexmed/qfaf024

**Published:** 2025-05-13

**Authors:** Daosheng Luo, Jintao Guo, Tongwen Chen, Guihua Liu, Peng Luo, Zhiquan Deng, Yu Xin Tang, Yongbin Liao, Chunhua Deng

**Affiliations:** Department of Urology, Dongguan People's Hospital, Dongguan 523000, China; Reproductive Medicine Centre, The Sixth Affiliated Hospital, Sun Yat-Sen University, Guangzhou 510000, China; Depatment of Urology, Zhuhai People's Hospital Medical Group, Zhuhai, Guangdong, 519000, China; Reproductive Medicine Centre, The Sixth Affiliated Hospital, Sun Yat-Sen University, Guangzhou 510000, China; Department of Urology, The First Affiliated Hospital of Sun Yat-sen University, Guangzhou, Guangdong 510080, China; Biomedical Innovation Center, The Sixth Affiliated Hospital, Sun Yat-sen University, Guangzhou 510000, China; Department of Urology, Dongguan People's Hospital, Dongguan 523000, China; Department of Urology, The Fifth Affiliated Hospital of Sun Yat-sen University, ZhuHai 519000, China; Department of Urology, Jiangmen Central Hospital, Jiangmen 529000, China; Department of Urology, The First Affiliated Hospital of Sun Yat-sen University, Guangzhou, Guangdong 510080, China; Biomedical Innovation Center, The Sixth Affiliated Hospital, Sun Yat-sen University, Guangzhou 510000, China

**Keywords:** chronic prostatitis/chronic pelvic pain syndrome, erectile dysfunction, Ningmitai capsule, Sildenafil

## Abstract

**Background:**

A high proportion of men with chronic prostatitis/chronic pelvic pain syndrome (CP/CPPS) present with comorbid erectile dysfunction (ED), but evidence-based therapeutic interventions specifically targeting this patient population remain understudied in clinical trials.

**Aim:**

To assess the efficacy of Ningmitai capsule (NMT), an oral traditional Chinese herbal formulation, combined with sildenafil versus monotherapy in alleviating symptoms among a cohort of participants with CP/CPPS and ED.

**Methods:**

A multi-center, randomized clinical trial was conducted from March 2019 to December 2022 at six tertiary hospitals in China. A total of 214 participants diagnosed with CP/CPPS and ED were randomized 1:2:2 to receive orally sildenafil (25 mg, q.n.), NMT (0.38 g × 4 capsules, t.i.d.), or a combination of both for 4 weeks. Validated Chinese version of the National Institutes of Health Chronic Prostatitis Symptom Index (NIH-CPSI), the International Index of Erectile Function-5 (IIEF-5) and the Erection Hardness Score (EHS) questionnaires were administered at baseline, week 2, and week 4.

**Outcomes:**

The primary endpoint was the reduction in NIH-CPSI pain domain scores from baseline to week 4.

**Results:**

All treatment groups exhibited statistically significant decreases in NIH-CPSI total, pain, urinary and quality of life (QoL) domain scores within 2 weeks, with improvements sustained until the end of the treatment. The combination group demonstrated superior pain score reductions versus sildenafil monotherapy at both timepoints (week 2: mean difference [MD] -2.82 ± 3.27 vs. -1.26 ± 3.45, *P* = 0.043; week 4, MD -3.57 ± 3.50 vs. -1.07 ± 2.94, *P* = 0.009). Notably, combination therapy achieved greater IIEF-5 score enhancements compared to NMT alone (*P <* 0.05) and higher responder rates than either sildenafil or NMT monotherapy (*P <* 0.05). No significant differences were found among the three arms concerning EHS. No adverse events were reported.

**Clinical Implications:**

NMT-sildenafil combination therapy may serve as a viable alternative to α-blocker-based regimens for CP/CPPS-ED patients, potentially circumventing the orthostatic hypotension risk associated with the concurrent use of phosphodiesterase 5 inhibitors (PDE5i) and α-blockers.

**Strengths and Limitations:**

Strengths include a prospective randomized design, which is well controlled. Limitations encompass the absence of placebo control and long-term follow-up.

**Conclusion:**

NMT-sildenafil combination therapy demonstrates significantly greater benefits of ameliorating pain symptoms and improving erectile function in men with CP/CPPS and ED compared to either monotherapy, with favorable tolerability profiles.

**Registration:**

The study protocol was reviewed and approved by the institutional ethics committee and was registered at ClinicalTrials.gov (NCT06064448).

## Introduction

Chronic prostatitis/chronic pelvic pain syndrome (CP/CPPS) represents a prevalent urogenital disorder affecting predominantly young and middle-aged men, with an estimated global prevalence of 8% to 10% among adult males. Clinically, over 90% of chronic prostatitis cases manifest as CP/CPPS, primarily characterized by persistent pelvic pain or discomfort lasting more than 3 months.^1-3^

CP/CPPS patients are more prone to cause sexual dysfunction than the general population, including erectile dysfunction (ED).^4^ A meta-analysis of 6615 patients revealed that ~62% of CP/CPPS patients present with some form of sexual dysfunction,^5^ while Chinese epidemiological data specifically report a 35.1% co-occurrence rate of ED in this population.^6^ These findings underscore the clinical imperative for integrated management regimes addressing both conditions.

Current first-line therapies present therapeutic challenges. α-blockers, which alleviate dysuria and other symptoms by antagonizing α_1_-adrenoceptors in the prostate and promoting relaxation of prostate smooth muscle, frequently induce urogenital adverse effects including abnormal ejaculation. Phosphodiesterase 5 inhibitors (PDE5i) are currently regarded as the primary treatment option for ED in CP/CPPS,^7^ and have been reported to alleviate CP/CPPS symptoms.^8^ Nevertheless, the concomitant use of PDE5i and α-blockers raises safety concerns regarding symptomatic hypotension and central nervous system effects (eg, headache and dizziness).^9^^,^^10^ This therapeutic dilemma necessitates exploration of alternative regimens with improved safety profiles.

Traditional Chinese medicine (TCM), which utilizes plant-derived or herbal products, has increased in popularity in both Asian and Western countries as an alternative and effective treatment for CP/CPPS due to its advantages including unique mechanisms of action which arise from its multi-component, multi-target and multi-mechanism nature, as well as its typically low side-effect profile.^11^^,^^12^ The Ningmitai capsule (NMT), a Chinese National Medical Products Administration approved TCM formula (Approval No. Z20025442) comprising seven herbal plants, has proven to be clinical efficacy in patients suffering from CP/CPPS in randomized controlled trials. Notably, a double blind study documented its superiority over placebo in alleviating CP/CPPS-related pain and enhancing quality of life,^13^ while subsequent research suggested synergistic effects on erectile function when combined with antibiotics.^14^ These findings position NMT as a guideline-recommended therapy in China.^2^^,^^15^

To address current therapeutic challenges, we performed a multicenter, randomized controlled trial evaluating three therapeutic approaches: sildenafil, NMT alone, and their combination. The primary aim of this study was to address the following question: Does the combination therapy achieve superior efficacy in reducing NIH-CPSI pain subdomain scores compared to monotherapies at 4-week treatment endpoint, while maintaining comparable safety? The secondary objectives included addressing the following question: Can the combination strategy achieve synergistic enhancement of erectile function beyond monotherapeutic effects in this comorbid population?

## Materials and methods

### Study design

This prospective, multicenter, randomized controlled study was conducted in the outpatient urology or andrology clinic of six tertiary hospitals in China. The study protocol was reviewed and approved by the institutional ethics committee (Ethical Approval Number: 201908A4) and was registered at ClinicalTrials.gov (NCT06064448). The trial was conducted in accordance with national Good Clinical Practice (GCP) guidelines, as well as the Declaration of Helsinki of the World Medical Association. All individual participants provided written informed consent prior to the initiation of any study-specific procedures. The reporting of this trial complied with the Consolidated Standards of Reporting Trials reporting guideline.

### Participants

Patients aged between 20 and 50 years with complaints of pelvic pain or discomfort for at least 3 months during the previous 6 months were recruited for the study. The diagnosis of CP/CPPS was established according to the National Institutes of Health (NIH) criteria^16^ and Chinese Urological Association guidelines.^2^ Men were eligible if they reported a pain sub-score of at least 4 or a urinary sub-score exceeding 4 on the NIH-CPSI, were sexually active with a female partner, and experienced ED, as indicated by an IIEF-5 score of no more than 21 points.

We excluded males with urogenital infections, bladder outlet obstruction, overactive bladder, varicoceles, severe organic ED, drug-induced or traumatic ED, abnormal external genitalia, or abnormal sex hormone levels. Additionally, we excluded individuals with a history of genitourinary cancer, prostate or bladder surgery, as well as those with severe heart, respiratory, liver, hematopoietic diseases, or renal dysfunction. Patients who had utilized medical therapies for CP/CPPS or ED, including antibiotics, α-blockers, PDE5i, or various TCMs or phytomedicines within the previous 2 weeks were also excluded.

### Randomization and allocation concealment

All eligible patients were randomly allocated in a 1:2:2 ratio to receive sildenafil, NMT or sildenafil plus NMT. The randomization was performed using a computer-generated random sequence with permuted blocks of size 6, stratified by site. Allocation concealment was conducted using opaque sealed envelopes. The clinicians were unaware of the group assignments before allocation to avoid selection of treatment. Although double blinding was not conducted in the study due to difficulties in placebo preparation, the statisticians remained blind to subject-group allocation.

### Interventions

Men were randomly assigned to receive oral sildenafil citrate tablets 25 mg nightly at bedtime, oral Ningmitai capsules (Guiyang Xintian Pharmaceutical Co., Ltd.) 0.38 g × 4 capsules three times daily, or a combination of both for 4 consecutive weeks. NMT is formulated in accordance with the traditional Chinese medicine compatibility principle of “sovereign, minister, assistant, and guide”, composed of the following botanical constituents: *Polygonum capitatum* Buch.-Ham. ex D. Don Prodr (Touhualiao, 30%), *Imperata cylindrica Beauv.* var. *major* (Nees) C. E. Hubb. (Baimaogen, 17%), *Cocculus orbiculatus* C. K. Schneid. (Dafengteng, 15%), *Berberis soulieana* Schneid. and *Berberis wilsonae* Hemsl. (Sankezhen, 11%), *Agrimonia pilosa* Ledeb. (Xianhecao, 11%), *Hibiscus mutabilis* L. (Mufurongye, 1%), and *Forsythia suspensa* (Thunb.) Vahl (Lianqiao, 15%). Each capsule complies with the Chinese national drug standard WS-10348-(ZD-0348)2002-2012Z, containing a minimum of 1 mg gallic acid as specified. HPLC analytical results demonstrate that the formulation contains multiple bioactive compounds, with principal active constituents including gallic acid, berberine, forsythoside A, rutin, and quercetin. Furthermore, patients were informed that the use of any other medications or therapies for the management of CP/CPPS or ED was prohibited throughout the study. If any alternative therapies were utilized, details were required to be documented.

### Measures and outcomes

All patients underwent a baseline assessment of CPPS symptoms using the NIH-CPSI, as well as evaluations of erectile function through the IIEF-5 and EHS. Subsequently, the enrolled patients were required to complete the NIH-CPSI, IIEF-5, and EHS questionnaires at two additional time points: midway through the trial (week 2) and at the end (week 4). Adverse events were monitored and recorded throughout the duration of the trial.

The NIH-CPSI (range 0–43) is a reliable and validated instrument for assessing CP/CPPS symptoms. It comprises three domains: pain (range 0–21), urinary symptoms (range 0-10), and QoL (range 0–12).^17^ Higher scores on the NIH-CPSI indicate worse conditions of CP/CPPS, with symptom severity classified as mild (0–14), moderate (15–29) or severe (30–43).^18^ The IIEF-5 (range 0–25) is a 5-item questionnaire, with each question scoring from 0 to 5, where lower scores indicate more severe dysfunction.^19^^,^^20^ The EHS is a validated single-item self-reporting outcome on the hardness of erection, ranging from I (soft penis) to IV (completely hard and fully rigid).^21^

The primary outcome of this study was the change in the NIH-CPSI pain score from baseline to week 4. The secondary outcomes included the NIH-CPSI total score, urinary and QoL domain scores, as well as the IIEF-5 and EHS scores, in addition to the response rate. Responders were defined as patients who achieved a reduction of at least 25% from baseline in the NIH-CPSI pain score, with or without an increase in either the IIEF-5 score or the EHS score. Participants for whom response assessments were unavailable were considered nonresponders and included in the denominator for the calculation of response rates. Additionally, a post hoc analysis were performed after study completion to explore the association between ED and NIH-CPSI total scores severity.

### Statistical analysis

The sample size calculations were based on an 80% power to determine whether the combination group was superior to the sildenafil group regarding the primary outcome. We assumed a 2-point difference between these two groups, although data on responses were limited for men diagnosed with CP/CPPS and ED. Therefore, a sample size of 61 in the combination group and 31 in the sildenafil group were required, following a 2:1 ratio, with a one-sided significance level (α) of 0.025. Considering a 20% dropout rate, a total of 193 participants were needed for this trial.

Efficacy analyses were based on the intention-to-treat (ITT) principle, incorporating all patients who underwent randomization. Statistical analyses were performed using SAS software version 9.4 (SAS Institute Inc., Cary, NC, USA), with a 2-sided *P* value of less than 0.05 considered statistically significant.

The comparison of NIH-CPSI scores and IIEF-5 scores between groups and the changes in scores were analyzed using one-way analysis of variance, and Bonferroni method was used to adjust the test level for pairwise comparisons among multiple groups. The comparison of EHS erection hardness between groups was conducted using the Kruskal-Wallis rank sum test, and the comparison of different visit periods within a group was performed using the Friedman rank sum test. Response rates were compared between groups using the chi-square test or Fisher’s exact probability method. The comparison of median IIEF-5 scores among three severity groups of NIH-CPSI total scores was analyzed using Kruskal-Wallis rank sum test.

## Result

### Patient demographics

**Figure 1:**
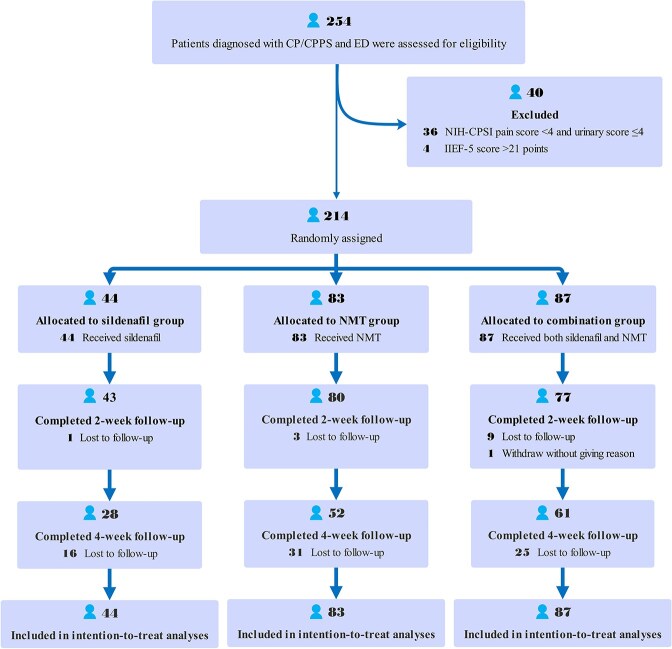
Participant flow diagram.

Between March 2019 and December 2022, a total of 254 patients with CP/CPPS were screened for eligibility at six clinical sites, 214 patients were enrolled, with 44 randomly assigned to receive sildenafil, 83 to receive NMT, and 87 to receive a combination of both (Figure 1). Notably, 89.4% of the enrolled patients were aged 40 years or younger. All patients allocated were included in the ITT analyses. Ultimately, 210 individuals completed the 2-week follow-up, while 141 patients completed the 4-week follow-up (Figure 1). There was no statistically significant difference in the completion rates at the end of the trial among the three groups (*P* = 0.483). Patients withdrawal or loss to follow-up was primarily attributed to the COVID-19 pandemic.

As shown in Table 1, all baseline characteristics were comparable among the three groups. The mean total and pain domain NIH-CPSI scores were 20.24 ± 6.94 and 7.13 ± 3.83, respectively. Nearly 80% of patients presented moderate to severe symptoms on the NIH-CPSI. The baseline scores for the NIH-CPSI total and three domains, as well as the IIEF-5, were similar across the treatment groups.

**Table 1:** Demographic and baseline characteristics.

Characteristics	Sildenafil Group	NMT Group	Combination Group	*P*
**Age (N, %)^a^**				0.447
20 ~ 25	7 (16.67)	17 (20.48)	9 (10.98)	
26 ~ 30	13 (30.95)	24 (28.92)	27 (32.93)	
31 ~ 40	15 (35.71)	34 (40.96)	39 (47.56)	
41 ~ 50	7 (16.67)	8 (9.64)	7 (8.54)	
**NIH-CPSI Score, Mean ± SD**				
Total Score	18.86$\pm$7.30	21.28$\pm$6.76	19.95$\pm$6.87	0.221
Pain Score	6.39$\pm$3.32	7.02$\pm$4.05	7.60$\pm$3.82	0.052
Urinary Score	4.84$\pm$2.80	5.55$\pm$2.47	4.61$\pm$2.57	0.305
Quality-of-Life Score	8.68$\pm$3.08	9.06$\pm$2.86	8.39$\pm$2.66	0.155
**Severity of NIH-CPSI score, N(%)**				0.108
Mild (0 ~ 14)	14 (31.82)	10 (12.05)	20 (22.99)	
Moderate (15 ~ 29)	26 (59.09)	61 (73.49)	57 (65.52)	
Severe (30 ~ 43)	4 (9.09)	12 (14.46)	10 (11.49)	
**IIEF-5 Score, Mean ± SD**	10.41$\pm$5.38	12.47$\pm$4.73	11.28$\pm$5.34	0.080
**Severity of IIEF-5 score, N(%)**				0.657
Mild (17 ~ 21)	5 (11.36)	18 (21.69)	17 (19.54)	
Mild to moderate (12 ~ 16)	16 (36.36)	35 (42.17)	31 (35.63)	
Moderate (8 ~ 11)	10 (22.73)	14 (16.87)	18 (20.69)	
Severe (0 ~ 7)	13 (29.55)	16 (19.28)	21 (24.14)	
**EHS Score, N(%)**				0.401
I	5 (11.36)	4 (4.82)	3 (3.45)	
II	16 (36.36)	22 (26.51)	27 (31.03)	
III	23 (52.27)	56 (67.47)	56 (64.37)	
IV	0 (0)	1 (1.20)	1 (1.15)	

Abbreviations: NMT, Ningmitai capsule; NIH-CPSI, National Institutes of Health Chronic Prostatitis Symptom Index; SD, standard deviation; IIEF-5, International Index of Erectile Function-5; EHS, Erection Hardness Score. a, age data were missing for seven subjects.

Furthermore, we analyzed the mean IIEF-5 scores of patients categorized by mild, moderate, and severe symptoms according to the NIH-CPSI score, and observed a statistically significant difference (*P* = 0.0078) (Table 2), indicating that more severe symptoms based on the NIH-CPSI score correlate with greater severity of ED. In contrast, no statistically significant difference was found among the mean NIH-CPSI total scores of subjects with varying degrees of ED (*P* = 0.0538), as detailed in Supplementary Table 1.

**Table 2:** The effect of NIH-CPSI level on mean IIEF-5 score.

	**N**	**IIEF-5 score (Mean ± SD)**	***P***
**Severity of NIH-CPSI score**			0.0078
Mild (0 ~ 14)	44	13.36$\pm$4.54	
Moderate (15 ~ 29)	144	11.38$\pm$5.10	
Severe (30 ~ 43)	26	9.54$\pm$5.67	
**Overall**	214	11.56$\pm$5.16	

Abbreviations: NIH-CPSI, National Institutes of Health Chronic Prostatitis Symptom Index; IIEF-5, International Index of Erectile Function-5.

### Study end points

We observed a statistically significant reduction in NIH-CPSI pain, urinary, QoL and total scores after 2 weeks of treatment within each therapy group (all *P <* 0.05). Table 3 summarizes the changes for all NIH-CPSI scores and IIEF-5 score from baseline to weeks 2 and 4. The average decreases from baseline for the NIH-CPSI pain domain score in the combination group were 2.82 points at week 2 and 3.57 points at week 4, both of which were significantly greater than the decrease observed in the sildenafil group (*P* = 0.043 and *P* = 0.009, respectively). There were no significant differences between the combination group and the sildenafil group in the changes of the other NIH-CPSI domains and the total score from baseline to weeks 2 and 4 (*P* > 0.05 for all comparisons), although the NIH-CPSI total score in the combination group at week 4 decreased by an average of 8.20 points, nearly twice the decrease seen in the sildenafil group (4.68 points). For the comparison between combination therapy and NMT monotherapy, no significant differences were observed in the change of NIH-CPSI pain, urinary, QoL and total scores at both 2 and 4 weeks (*P* > 0.05 for all comparisons).

**Table 3:** Comparison of changes in NIH-CPSI and IIEF-5 scores after 2 and 4 weeks of treatment.

	Sildenafil Group	NMT Group	Combination Group	*P*	*P_NMT vs sildenafil_*	*P_combination vs sildenafil_*	*P_combination vs NMT_*
**NIH-CPSI score**							
** Pain score**							
2 weeks	−1.26 ± 3.45	−2.35 ± 3.30	−2.82 ± 3.27	0.049	0.249	**0.043**	1.000
4 weeks	−1.07 ± 2.94	−3.00 ± 4.10	−3.57 ± 3.50	**0.012**	0.076	**0.009**	1.000
** Urinary score**							
2 weeks	−1.07 ± 2.02	−1.95 ± 2.34	−1.16 ± 2.35	**0.044**	0.127	1.000	0.090
4 weeks	−1.57 ± 2.60	−2.00 ± 2.28	−1.72 ± 2.68	0.736	1.000	1.000	1.000
** QoL score**							
2 weeks	−1.21 ± 3.33	−1.86 ± 3.15	−1.94 ± 2.61	0.407	0.751	0.615	1.000
4 weeks	−2.04 ± 3.40	−2.46 ± 4.67	−2.90 ± 3.28	0.602	1.000	0.988	1.000
** Total score**							
2 weeks	−2.53 ± 5.56	−5.79 ± 6.48	−5.16 ± 6.18	**0.019**	**0.018**	0.081	1.000
4 weeks	−4.68 ± 6.52	−7.46 ± 9.40	−8.20 ± 6.71	0.139	0.388	0.149	1.000
** IIEF-5 score**							
2 weeks	1.28 ± 4.92	−0.36 ± 5.77	2.30 ± 5.38	**0.010**	0.338	0.980	**0.008**
4 weeks	1.54 ± 5.65	0.21 ± 6.52	3.56 ± 6.32	**0.019**	1.000	0.480	**0.016**

Abbreviations: NMT, Ningmitai capsule; NIH-CPSI, National Institutes of Health Chronic Prostatitis Symptom Index; IIEF-5, International Index of Erectile Function-5; EHS, Erection Hardness Score.

The mean improvements in the IIEF-5 score from baseline for the combination group at weeks 2 and 4 were 2.30 and 3.56, respectively, both of which were significantly greater versus the NMT group (*P* = 008 and *P* = 0.016, respectively); however, no significant differences were found when compared to the sildenafil group (*P* = 0.980 and *P* = 0.480, respectively).

**Figure 2:**
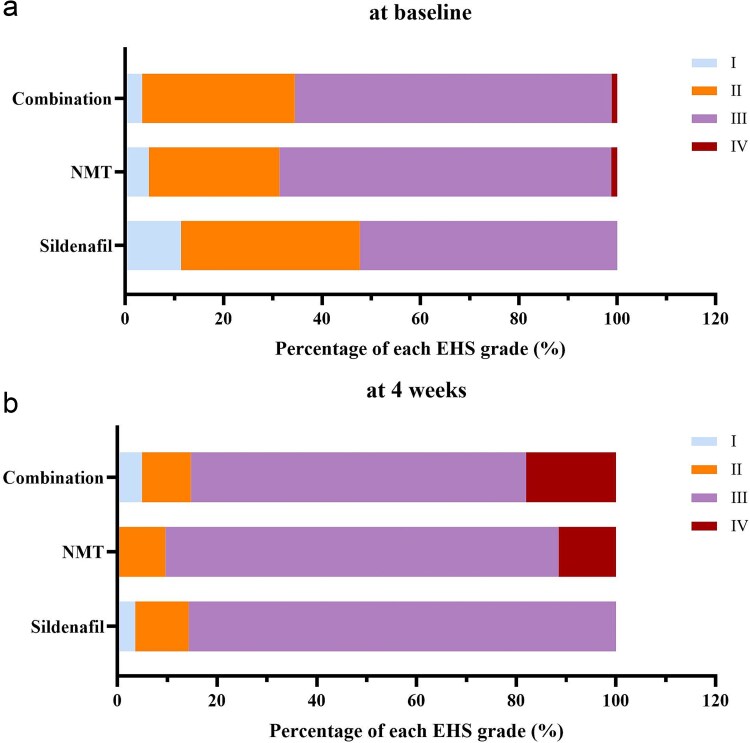
The EHS grade distribution at baseline (a) and at the end of 4-week treatment (b).

The distribution of EHS grades at baseline and at the end of the trail is illustrated in Figure 2. The proportion of EHS grade III or IV were increased in all three groups, but no significant differences were observed among the three arms (*P* > 0.05 for all comparison).

The comparison of responders is shown in Table 4. At week 4, 45 patients (51.72%) in the combination group, 13 patients (29.55%) in the sildenafil group, and 32 patients (38.55%) in the NMT group experienced at least a 25% decline in NIH-CPSI pain score compared to baseline (CPPS symptom responders). A significant difference in CPPS symptom responders was observed in favour of combination therapy compared with sildenafil (*P* = 0.016); however, no significant difference was found between combination and NMT groups (*P* = 0.085). The proportion of men with a ≥ 25% decrease in NIH-CPSI pain domain and any increase in IIEF-5 or EHS (CPPS symptom and erectile function co-responders) was 43.68% in the combination group compared to 22.73% in the sildenafil group and 28.92% in the NMT group, indicating that combination therapy resulted in significantly greater improvements compared with both sildenafil (*P* = 0.019) and NMT (*P* = 0.046) monotherapy.

**Table 4:** Comparison of response rate at the end of 4-week treatment.

	**Sildenafil Group**	**NMT Group**	**Combination Group**	**NMT *vs.* Sildenafil**		**Combination *vs.* Sildenafil**		**Combination *vs.* NMT**	
				**Difference (95%*CI)***	***P***	**Difference (95%*CI)***	***P***	**Difference (95%*CI)***	***P***
**Responders, n(%)**									
≥ 25% decrease in NIH-CPSI pain score	13(29.55%)	32(38.55%)	45(51.72%)	9.01(-8.06, 26.08)	0.313	22.18(5.09,39.27)	**0.016**	13.17(-1.66,28.00)	0.085
≥ 25% decrease in NIH-CPSI pain score and any increase in IIEF-5 or EHS	10(22.73%)	24(28.92%)	38(43.68%)	6.19(-9.57,21.95)	0.454	20.95(4.77,37.14)	**0.019**	14.76(0.49,29.04)	**0.046**

Abbreviations: NMT, Ningmitai capsule; CI, confidence interval; NIH-CPSI, National Institutes of Health Chronic Prostatitis Symptom Index; IIEF-5, International Index of Erectile Function-5.

No drug-related adverse events or serious adverse events were reported during the study. All regimens were well tolerated, and the simultaneous administration of both drugs did not increase the incidence of adverse events.

## Discussion

In 1981, Keltikangas-Jarvinen et al.^22^ evaluated patients suffering from chronic prostatitis using a psychiatric interview, revealing that 52% of the 42 participants reported experiencing periodic sexual dysfunction. Since then, the clinical association between chronic pelvic pain and sexual dysfunction has been repeatedly demonstrated.^6^^,^^23^^,^^24^ A meta-analysis of 24 studies involving 11 189 men reported that the overall prevalence of sexual dysfunction in men with CP/CPPS was 62%, with a specific prevalence of ED at 29%.^5^ ED has been recognized to be more prevalent among patients with pelvic pain compared to the general population.^25^

In this study, we found that patients with more severe prostatitis symptoms tend to have lower IIEF-5 score which means poorer erectile function. As in our study, Tan et al. conducted a population-based survey in Singapore involving 1087 men, which revealed that those with prostatitis-like symptoms had worse erectile function than those without prostatitis-like symptoms (IIEF-5 score 11.92 vs. 17.16, *P <* 0.003).^26^ Similarly, Marszalek et al. also observed in an epidemiological study of 1765 individuals that chronic pelvic pain symptoms (measured by NIH-CPSI) negatively impacts erectile function (measured by IIEF-5).^27^

In brief, more severe CP/CPPS is related not only to higher prevalence of ED, but also to greater ED severity based on IIEF scores. As such, some researchers proposed the addition of sexual dysfunction to the UPOINT system, which is employed to guide the treatment of CP/CPPS.^28^^,^^29^ Although this proposal is controversial,^30^^,^^31^ it highlights the significance of assessing and appropriately treating ED in patients with CP/CPPS.

While it is well-established that CP/CPPS is associated with ED, the underlying mechanisms remain unclear. Factors such as mental distress, endocrine and neurological influences, as well as impaired QoLrelated to the illness may contribute to the sexual dysfunction observed in patients with CPPS, but the presence of erectile disorders is more frequently associated with symptoms indicative of a more severe inflammatory condition.^25^^,^^32^

PDE5is facilitate smooth muscle relaxation within the prostate through the modulation of nitric oxide (NO)-cyclic guanosine monophosphate (cGMP) signaling, thereby alleviating CP/CPPS-related pain by inhibiting the inflammatory pathway and reducing intraprostatic pressure.^8^^,^^33^ Daily sildenafil significantly enhances penile blood flow and penile endothelial function.^34^ Taking sildenafil at bedtime results in longer-lasting benefits on endothelial function^35^ and enhances nocturnal erections in both healthy men and those with ED, contributing to the maintenance of the morphodynamic integrity of smooth muscle cells within the corpora cavernosa.^36^^,^^37^ However, the effect of sildenafil on enhancing blood flow in the cavernosum may lead to high doses exacerbating local inflammation and swelling in patients with prostatitis, potentially impeding the resolution of prostate inflammation. Our selection of low-dose sildenafil (25 mg) balanced these potential benefits against concerns regarding prostatic vascular engorgement exacerbating inflammation. Consistent with previous studies,^8^^,^^38^ our data revealed that nightly sildenafil use alleviated CPPS symptoms.

However, these improvements appears to be limited. This may be mainly attributed to the fact that CP/CPPS is a heterogeneous syndrome with a multifactorial etiology. For ED induced by CP/CPPS, treatment should prioritize addressing the underlying CPPS. Rather than employing a single treatment for multiple symptoms, a multimodal approach is recommended. Chinese herbal medicine is expected to treat patients’symptoms comprehensively and can serve as a significant component of a multimodal therapeutic plan.^39^

The rationale for combining NMT, a Chinese patent medicine based on traditional Chinese medicine theory, with sildenafil stems from their complementary mechanisms. Network pharmacological analyses identify over 47 bioactive components in NMT, including quercetin, kaempferol, and β-sitosterol, which modulate PI3K/AKT and MAPK signaling to suppress proinflammatory cytokine production.^40^ Notably, quercetin, an herbal anti-inflammatory and antioxidant supplement, with proven symptomatic amelioration in CP/CPPS in a small randomized controlled trial,^41^^,^^42^ also showed improvement in ED in an animal model.^43^ Chen and Liu et al.^44^^,^^45^ revealed the anti-inflammatory, antioxidative, and pain-relieving effects of NMT on autoimmune prostatitis in two different animal models. NMT suppressed CCL2-MAPK, NF-κB, and STAT3, while also reducing substance *P* expression in the dorsal root ganglia in CPPS animal models, indicating that multi-pathways and multi-targets are implicated in its therapeutic action. A detailed description of the pharmacological effects of NMT is presented in Table 5. In addition, a 4-week prospective, double-blind, placebo-controlled trial (n = 120) reported significant NIH-CPSI improvements with NMT monotherapy.^13^ Our combination strategy builds upon these findings by simultaneously addressing inflammatory pain (via NMT) and microcirculatory dysfunction (via PDE5i).

**Table 5:** Summary of pharmacological effects, active ingredients and mechanisms of Ningmitai capsule.

Pharmacology	Experimental Subject	Effects	Active ingredient	Source drug	Ref
Anti-inflammatory	experimental autoimmune prostatitis (EAP) rat models non-obese diabetes-experimental autoimmune prostatitis (NOD-EAP) mouse model	Decrease: MAPK, NF-κB, STAT3, TNF-α, IL-1α, IL-1β, IL-6, IL-17A, IL-23, IL-27, INF-β、MCP-1	Quercetin, Kaempferol	*Polygonum capitatum* Buch.-Ham. ex D. Don, *Agrimonia pilosa* Ledeb., *Hibiscus mutabilis* L., *Forsythia suspensa (Thunb.)* Vahl.	^40^ ^,^ ^44^ ^,^ ^45^
			Gallic acid	*P. capitatum* Buch.-Ham. ex D. Don	
			Berberine	*Berberis soulieana* C.K.Schneid.	
			Beta-sitosterol	*Cocculus orbiculatus* C.K.Schneid.	
			Rutin, Hyperin	*H. mutabilis* L.	
Pain-relieving	experimental autoimmune prostatitis (EAP) rat models non-obese diabetes-experimental autoimmune prostatitis (NOD-EAP) mouse model	Decrease: substance *P;* CCL2; GFAP	Quercetin	*P. capitatum* Buch.-Ham. ex D. Don, *Agrimonia pilosa* Ledeb., *H. mutabilis* L. *F. suspensa (Thunb.)* Vahl.	^40^ ^,^ ^44^ ^,^ ^45^
			Rutin, Hyperin	*H. mutabilis* L.	
Antioxidant	experimental autoimmune prostatitis (EAP) rat models	Increase: SOD Decrease: MDA	Quercetin, Kaempferol	*P. capitatum* Buch.-Ham. ex D. Don, *Agrimonia pilosa* Ledeb., *H. mutabilis* L. *F. suspensa (Thunb.)* Vahl.	^40^ ^,^ ^44^
Antispasmodic	Isolated Smooth Muscle from Rat Bladder Isolated Rabbit Urethral Smooth Muscle	Relaxes the smooth muscles of the bladder and urethra, reduces spasms, and regulates the function of the bladder detrusor and sphincter.	Quercetin	*P. capitatum* Buch.-Ham. ex D. Don, *Agrimonia pilosa* Ledeb., *H. mutabilis* L., *F. suspensa (Thunb.)* Vahl.	^46-48^
			Berberine	*Berberis soulieana* C.K.Schneid.	
			PHILLYRIN	*F. suspensa (Thunb.)* Vahl.	
Diuresis	Normal rat, rabbit, dog	Increase: Urinary volume	Gallic acid	*P. capitatum* Buch.-Ham. ex D. Don	^49^ ^,^ ^50^
			Berberine	*Berberis soulieana* C.K.Schneid.	
Antibacterial	*Staphylococcus epidermidis* *Staphylococcus aureus* *Escherichia coli* *Candida albicans*	Decrease: Proliferation and its biofilm formation, serp2195, gpxA-2	Gallic acid	*P. capitatum* Buch.-Ham. ex D. Don	^51^
			Quercetin	*P. capitatum* Buch.-Ham. ex D. Don, *Agrimonia pilosa* Ledeb., *H. mutabilis* L., *F. suspensa (Thunb.)* Vahl.	
			Berberine	*Berberis soulieana* C.K.Schneid.	
			Luteolin	*P. capitatum* Buch.-Ham. ex D. Don, *Agrimonia pilosa* Ledeb, *F. suspensa (Thunb.)* Vahl.	

Pain has been recognized to impact more on QoL than urinary symptoms. The level of pelvic pain in men affected by CP/CPPS is strongly associated with sexual dysfunction.^32^ Therefore, we assessed the change in the NIH-CPSI pain score as the primary outcome of this study. Our data showed the combination therapy resulted in a more significant improvement in NIH-CPSI pain scores compared to sildenafil monotherapy at both weeks 2 and 4. Additionally, after 4 weeks of treatment, the NIH-CPSI total score for patients receiving combination therapy decreased by an average of over 8 points. This degree of change exceeded the minimum clinically significant difference of 4-6 points, which patients perceive as beneficial, indicating that the results are clinically meaningful.^52^^,^^53^ Furthermore, the responder rates observed also favor combination therapy in comparison to sildenafil or NMT monotherapy. These results suggest that while NMT alone effectively addresses pain symptom, the addition of sildenafil may better address the multifactorial pathophysiology of CPPS-ED comorbidity through PDE5i’s proposed mechanisms in pelvic microcirculation and smooth muscle modulation. However, about half of the patients did not respond, which may be related to the short course of treatment.

Finally, NMT demonstrated a favorable safety profile, consistent with its post-marketing surveillance data showing rare adverse reactions, primarily mild gastrointestinal symptoms (eg, nausea, vomiting, abdominal discomfort). A toxicology study in GLP-certified institution further supported its safety: a 26-week repeated-dose study in rats revealed no significant toxicity, with a NOAEL of 3.6 g/kg (~47 times the clinical dose), confirming its wide therapeutic margin. Notably, no drug-related adverse or serious adverse events were reported during the trial. This absence of reported toxicity may be attributed to multiple factors: (1) Enhanced pharmacological tolerance in chronic patients with prior PDE5i exposure; (2) Conservative sildenafil dosing; (3) Limited power for rare event detection. Importantly, the combination therapy exhibited favorable tolerability without evidence of pharmacodynamic interactions. Of particular clinical relevance, we observed no instances of orthostatic hypotension—a recognized safety concern when combining PDE5is with α-blockers. This finding suggests that NMT may possess distinct interaction profiles compared to conventional α-blockers, potentially offering safer combination options with sildenafil for CPPS-ED management. However, comprehensive pharmacokinetic studies are required to elucidate potential herb-drug interactions.

This study has several limitations that warrant consideration. First, the absence of a placebo control precludes the determination of true efficacy after accounting for the placebo effect. Second, we looked at only a fixed daily dosage of a single PDE5i; using a variable dosage of the oral PDE5i or a long-acting PDE5i such as tadalafil may be a more effective component of a multimodal therapy. Third, a high rate of patient loss to follow-up, primarily due to the COVID-19 pandemic, may introduce selection bias and limit the generalizability of the findings. Fourth, the reliance on subjective endpoints constrain pathophysiological insights. Future trials should systematically integrate mechanism assessments to elucidate molecular pathways. Most importantly, the duration of this study was limited to 4 weeks, which is insufficient for the management of CPPS or ED, potentially underestimating long-term efficacy. Longer-term studies are needed to evaluate sustained efficacy.

## Conclusion

This preliminary investigation suggests that NMT-sildenafil combination therapy may synergistically improve CP/CPPS symptoms and erectile function through multi-target mechanisms. A larger, properly powered, placebo-controlled trial with rigorous design and long duration (≥ 12 weeks) is necessary to confirm this beneficial effect and to determine which patients would most benefit from it.

## Supplementary Material

Supplementary_Table_1_qfaf024
