# Supplementary material for: The efficacy of combination therapy with Ningmitai capsule and sildenafil in men with chronic prostatitis/chronic pelvic pain syndrome and erectile dysfunction: a prospective, multicenter, randomized controlled trial
Source: Sex Med. 2025 May 13;13(2):qfaf024. doi: 10.1093/sexmed/qfaf024 (PMC12074575; doi:10.1093/sexmed/qfaf024)
Supplement: Supplementary_Table_1_qfaf024 [file supplementary_table_1_qfaf024.docx]

**Supplementary Table 1.** The effect of IIEF-5 level on mean NIH-CPSI score.

|  | **N** | **NIH-CPSI score**  **(Mean±SD)** | *P* |
| --- | --- | --- | --- |
| **Severity of IIEF-5 score** |  |  | 0.0538 |
| Mild (17~21) | 40 | 18.78$\pm$6.22 |  |
| Mild to moderate (12~16) | 82 | 19.99$\pm$6.37 |  |
| Moderate (8~11) | 42 | 19.48$\pm$7.31 |  |
| Severe (0~7) | 50 | 22.48$\pm$7.71 |  |
| **All cases** | 214 | 20.24$\pm$6.94 |  |

Abbreviations: NIH-CPSI, National Institutes of Health Chronic Prostatitis Symptom Index; IIEF-5, International Index of Erectile Function-5.
